# Two distinct clinical patterns of checkpoint inhibitor-induced thyroid dysfunction

**DOI:** 10.1530/EC-19-0473

**Published:** 2020-03-11

**Authors:** Anna Olsson-Brown, Rosemary Lord, Joseph Sacco, Jonathan Wagg, Mark Coles, Munir Pirmohamed

**Affiliations:** 1 Department of Molecular and Clinical Pharmacology, University of Liverpool, Liverpool, UK; 2 The Clatterbridge Cancer Centre, Wirral, UK; 3 Molecular and Clinical Cancer Medicine, University of Liverpool, Liverpool, UK; 4 Roche Innovation Center, Basel, Switzerland; 5 Kennedy Institute of Rheumatology, University of Oxford, Oxford, UK

**Keywords:** immune related adverse events, thyroid dysfunction, checkpoint inhibitors, tumour immunotherapy

## Abstract

**Introduction:**

Immune checkpoint inhibitors can lead to thyroid dysfunction. However, the understanding of the clinical phenotype of ICI-induced thyroid dysfunction in the real-world population is limited. The purpose of this study was to characterise the clinical patterns of dysfunction and evaluate the demographic, biochemical and immunological features associated with this patient cohort.

**Materials and methods:**

To characterise the longitudinal clinical course of thyroid dysfunction in patients from a single, UK regional cancer centre, a retrospective review of patients was conducted. Inclusion criteria included all patients treated with antiPD-1 checkpoint inhibitors (ICI), either as monotherapy (pembrolizumab/nivolumab) or in combination with a CTLA-4 inhibitor (ipilimumab). Patterns of toxicity were evaluated together with assessment of antibody titres.

**Results:**

Over 16 months, thyroid dysfunction was seen in 13/90 and 3/13 patients treated with anti-PD1 monotherapy and in combination with ipilimumab, respectively. Patients either developed hyperthyroidism followed by hypothyroidism (12/16) or *de novo* hypothyroidism (4/16). Most patients were female (*n* = 11). All patients required thyroid replacement therapy. There was no relationship between clinical pattern of dysfunction and the presence of thyroid autoantibodies.

**Conclusions:**

There are two distinct patterns of thyroid dysfunction in ICI-treated patients. Patients with thyroiditis develop subsequent hypothyroidism in the vast majority of cases. The potential benefit from steroids or other therapy to manage the hyperthyroid phase remains unclear. Early detection of these patients through appropriate monitoring will improve clinical management and early hormone replacement, reducing the symptomatic burden of hypothyroidism.

## Introduction

Oncological immune checkpoint inhibitors (ICI) are transforming oncological practice (1, 2, 3). The first ICI, ipilimumab (Yervoy®), a CTLA-4 inhibitor, was used exclusively in metastatic malignant melanoma post-licensing (3, 4). Subsequently, the PD-1 ICIs, nivolumab (Opidivo®) and pembrolizumab (Keytruda®), have led to increased efficacy and, in general, a superior safety profile compared to CTLA-4 inhibitors (5). Efficacy can be further improved by combining nivolumab and ipilimumab (1, 2, 6). ICIs are now used in the mainstream treatment of metastatic melanoma, non-small cell lung cancer renal cell carcinoma, urothelial and head and neck cancers (7, 8). Nivolumab and pembrolizumab also hold global licences for additional indications including gastro-oesophageal adenocarcinoma, classical Hodgkin’s lymphoma and malignancies expressing microsatellite instability.

The use of ICIs has led to a range of novel adverse drug reactions (1) (hereafter called immune related adverse events (irAE)) which resemble endogenous autoimmune disease (9). Rash and colitis are the predominant irAEs caused by ipilimumab. The predominant endocrine irAE induced by ipilimumab is hypophysitis, occurring in 4–13% of patients (1, 2, 4). Thyroid dysfunction is witnessed in 1–5% of patients (1, 2, 4).

In the oncology setting, adverse events are graded using the Common Terminology Criteria for Adverse Events (CTCAE) criteria (10) from 1 to 5, with grade 1 representing mild toxicity and 5 representing death as a result of toxicity. As monotherapy, anti-PD-1 agents induce grade 3/4 (severe/life-threatening) irAEs in 15% of patients (1, 7). The incidence of severe irAEs increases further with combination checkpoint blockade with grade 3/4 irAEs occurring in 58–68% of patients (1, 2). The incidence of thyroid dysfunction is increased with PD-1 inhibitor therapy when compared with ipilimumab (6, 9, 10). While the majority of irAEs are treatable and reversible with corticosteroid containing immunosuppression, the endocrinopathies appear to be irreversible and, in the majority of cases, require lifelong replacement therapy (11).

Thyroid dysfunction reported within metastatic malignant melanoma clinical trials included both hyperthyroidism and hypothyroidism (Supplementary Table 1, see section on supplementary materials given at the end of this article) but the clinical and biochemical manifestations were not detailed. Radioisotope scanning of patients experiencing thyroid dysfunction has suggested a cytotoxic mechanism rather than autoantibody driven disease. Indeed, the association with autoantibodies remains unclear with variable results between studies (12, 13). Active management of ICI-induced thyroiditis is required despite the fact that the acute toxicity is of lower grade than other irAEs. It is also generally permanent, with only limited reports of recovery (14). No clinical predictors of thyroid toxicity following ICI therapy have been identified, and baseline TSH does not appear to predict the occurrence of thyroiditis (12). ICI treatment duration may be positively correlated with the occurrence of thyroiditis (15).

The aim of our paper was to characterise the longitudinal clinical course of thyroid dysfunction in patients from a single, UK regional cancer centre to determine if there is a characteristic pattern of biochemical abnormality, the proportion of patients experiencing clinically significant symptoms and the relationship of autoantibodies with biochemical change. Furthermore, we examined whether ypothyroidism was indeed the predominant manifestation of ICI-induced thyroid toxicity.

## Materials and methods

A retrospective review of case notes of patients treated with anti-PD-1 ICIs for metastatic melanoma over a 16-month treatment period was undertaken at the Clatterbridge Cancer Centre, UK. The review included all patients treated with anti-PD1 ICI, either as monotherapy (pembrolizumab/nivolumab) or nivolumab in combination with ipilimumab, an anti-CTLA4 ICI. Patient electronic records were reviewed to identify patients with thyroid dysfunction defined as the presence of deranged thyroid function denoted by a T4 level outside the standard reference range. Patients were considered to have had thyroid dysfunction irrespective of their clinical symptoms (i.e. clinical and subclinical biochemical disturbance). The presentation, clinical course (biochemical and symptomatic) and emerging longitudinal biochemical patterns of toxicity were evaluated to determine the presence of specific clinical trajectories within the cohort. All patients found to have a single abnormal T4 level were evaluated to determine if the changes were transient or led to established thyroid dysfunction. Within this cohort, all patients found to have a single abnormal reading went on to develop either HH or DN. Thyroid autoantibody titres (thyroid receptor antibody (TRAb), anti-thyroid peroxidase (TPO)) were also determined. Patients who had a diagnosis of pre-existing thyroid disorders (either hyper- or hypothyroidism) were excluded from the patient cohort. Aside from T4, if patients were found to have baseline biochemical parameters outside the normal ranges (eg TSH) but were subclinical and had no formal diagnosis of thyroid dysfunction, they were included in the cohort.

The local reference ranges for thyroid function tests were as follows: free T4 11.5–22.7 pmol/L; TSH 0.3–5.5 mU/L; free T3 3.1–7 pmol/L; TRAb 0.0–1.8 U/L and anti-TPO 0.0–33.9 IU/mL.

### Patient sampling and processing

As part of the standard clinical management of ICI administration at the Clatterbridge Cancer Centre, all patients’ thyroid status (TSH, T4) is assessed at baseline. Recurrent testing of TSH (with T4 and T3 evaluated if TSH is found to be outside the reference range) is then performed on a 3-weekly basis, alongside a standardised biochemical panel including full blood count, urea and electrolytes, liver function tests, cortisol and random glucose levels. Patients experiencing thyroid dysfunction also undergo thyroid autoantibody assessment. Anti-thyroglobulin antibodies are not analysed in routine clinical practice, and therefore all living patients with thyroid dysfunction during ICI therapy were approached to donate a blood sample in line with the Clatterbridge Biobank ethical approval. Informed consent was obtained. The presence of anti-Tg antibodies was analysed by ELISA using a human anti-thyroglobulin ELISA kit (ab178631) on serum isolated from donated samples.

### Data analysis

Time to event is presented as median durations with associated interquartile ranges. Descriptive statistics are presented as means with 95% CIs. Comparisons between groups have utilised Mann–Whitney and Kruskal3-weekly basisWallis analyses, as appropriate. All statistical tests utilised a significance value of *P* < 0.05.

## Results

### Patient demographics

Over the 16-month period, between February 2016 and May 2017, a total of 103 patients were treated with PD-1 ICIs for metastatic melanoma in the palliative setting. Ninety patients received monotherapy, while 13 received combination treatment. Thyroid dysfunction was observed in 16/103 of patients; 13 out of the 90 received monotherapy and 3/13 received combination therapy. All patients within the cohort were Caucasian (Table 1). For all other patients, thyroid function remained within the normal range throughout therapy.

**Table 1:** Patient demographics by treatment group and pattern of thyroid dysfunction.

Parameter	Monotherapy treatment group	Combination therapy treatment group	Hyperthyroidism followed by hypothyroidism (HH)	*De novo* hypothyroidism (DN)
Number of patients (*n* = 16)	13	3	12	4
Gender				
Male (M)	4	1	5	0
Female (F)	9	2	7	4
Gender differential of total treatment population (*n* = 103)	Males 57/103 Females 46/103	Males 57/103 Females 46/103	Males 57/103 Females 46/103	Males 57/103 Females 46/103
Age (years)				
Mean	58	57	56	70.5
Range	29–74	46–64	29–64	48–89
Ethnicity^a^				
White British	13/13	2/3	11/12	4/4
White Polish	0/13	1/3	1/12	0/4
Other	0/13	0	0/12	0/4
Melanoma subtype				
Cutaneous	11/13	2/3	8/12	4/4
Uveal	1/13	1/3	3/12	0/4
Anal	1/13	0/3	1/12	0/4
Number of metastatic sites				
1	4/13	1 (33.3%)	3/12	1/4
2	6/13	1 (33.3%)	6/12	2/4
≥3	3/13	1 (33.3%)	3/12	1 /4
Line of treatment				
1	5/13	1/3	5 /12	3/4
2	6/13	1/3	6/12	0/4
3	2/13	1/3	1/12	1/4
Type of treatment				
Monotherapy	13/13	3/3	9/12	4/4
Combination therapy	0/13	0/3	3/12	0/4

^a^Likely to reflect the ethic mix of the local population.

### Patterns of thyroid dysfunction and treatment

Two clear patterns of thyroid dysfunction were identified (Table 1): hyperthyroidism followed by hypothyroidism (HH) in 12/16 of cases and *de novo* (DN) hypothyroidism in 4/16 of cases.

**Figure 1:**
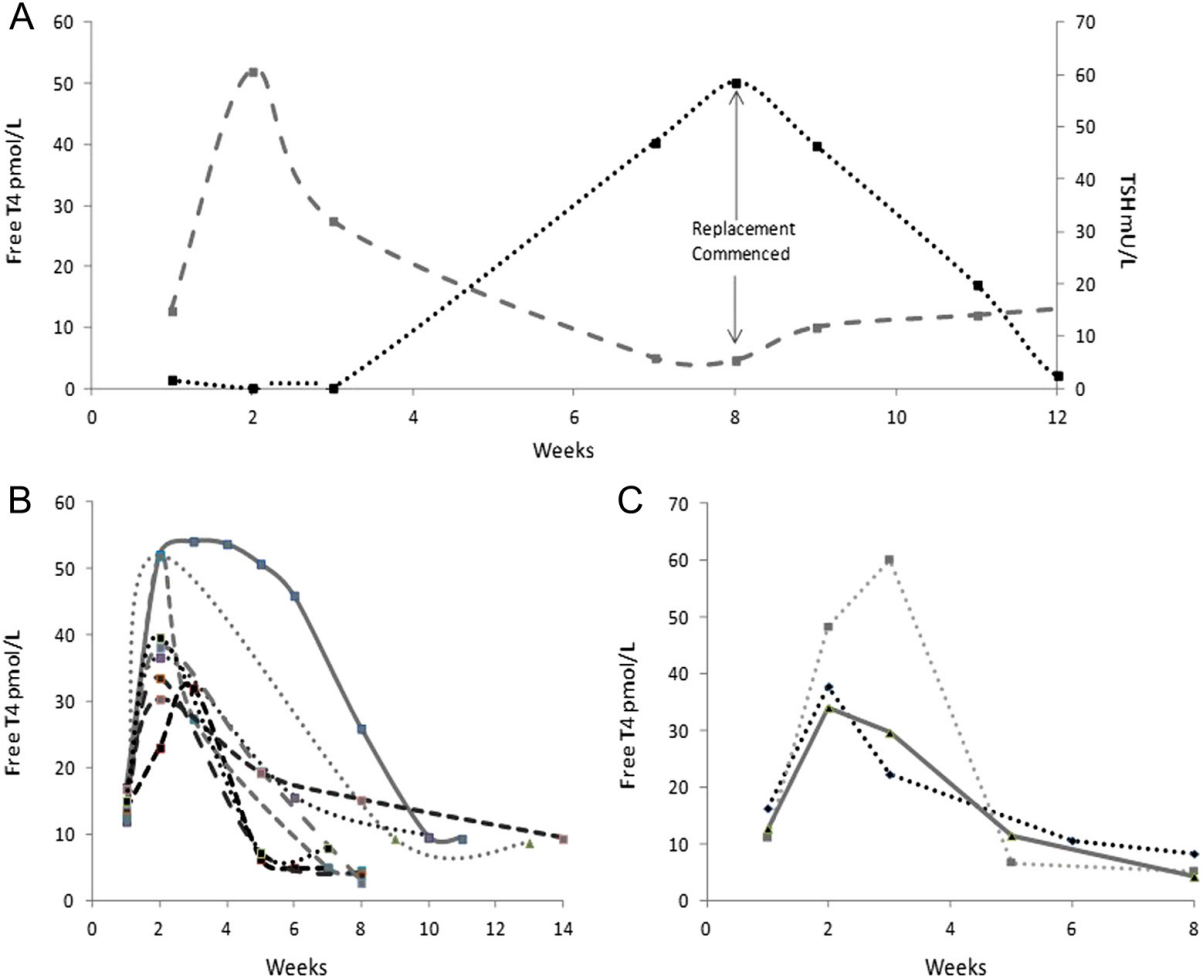
Changes in TSH (black) and T4 (grey) with the thyroid dysfunction characterised by an initial phase of hyperthyroidism followed by hypothyroidism (HH). (A) Changes in thyroid hormones over time. (B) Pattern of T4 changes from the point of dysfunction in patients receiving monotherapy immunotherapy. (C) Pattern of T4 changes from the point of dysfunction in patients receiving combination immunotherapy with ipilimumab and nivolumab.

With the HH pattern, there was an initial period of, minimally symptomatic or asymptomatic, hyperthyroidism followed by a decline in T4 and subsequent hypothyroidism. Where TSH was found to be low, both T4 and T3 were measured, but there were no cases of T3 elevation in the presence of a T4 within the normal range. The HH pattern was seen in 9/13 of patients receiving monotherapy and in all patients receiving combination therapy (Fig. 1A, B and C). The median time to onset was 9 weeks (range 6–27; IQR 3) and 3 weeks (range 3–6; IQR 3) with monotherapy ICI and combination therapy, respectively. The hyperthyroid phase duration was 9 weeks (range 3–15; IQR 6) in patients receiving monotherapy and universally 6 weeks (range 6; IQR 0 as) in those receiving combination therapy. The time to onset of thyroid dysfunction from the commencement of ICI therapy was significantly longer (*P* = 0.02) in the monotherapy group, but the duration of the hyperthyroid phase did not differ between monotherapy and combination therapy patients (*P* = 0.25). The T4 peak was 39.3 pmol/L (95% CI 32.7–45.9 pmol/L) and lasted 6–21 days. All cases were associated with a trough TSH of <0.1 mU/L. The mean trough T4 seen prior to thyroid replacement in the pooled analysis was 6.6 pmol/L (95% CI 4.9–8.3 pmol/L), associated with a mean peak TSH of 53.2 mU/L (95% CI 25.6–80.8 mU/L).

**Figure 2:**
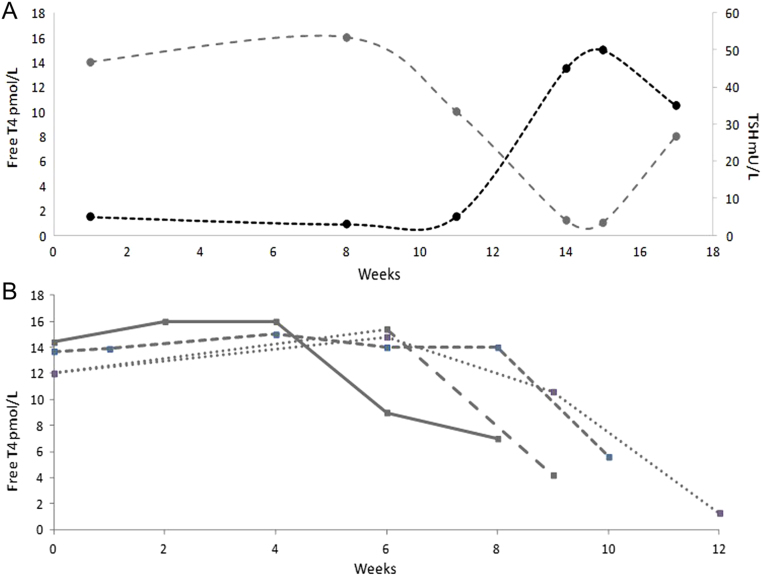
Changes of TSH (black) and T4 (grey) changes with the *de novo* (DN) pattern of thyroid dysfunction. (A) Changes in thyroid hormones over time. (B) Pattern of T4 changes from the point of dysfunction in patients receiving monotherapy immunotherapy.

The second pattern of thyroid dysfunction, DN hypothyroidism, did not have a preceding hyperthyroid phase (Fig. 2A and B). However, there were two patients who had TSH levels <0.3 mU/L but with normal T3/T4 during therapy. TSH levels were within the normal range (4.38 mU/L; 3.63 mU/L) at baseline in these two patients. All patients with DN hypothyroidism were asymptomatic prior to the detection of the hypothyroid state, apart from grade 1 fatigue in four patients. The median time to development of thyroid dysfunction after monotherapy ICI was 12 weeks (range 6–18 weeks; IQR 6 weeks). The trough T4 prior to commencement of levothyroxine was 4.45 pmol/L (95% CI 0.4–8.5 pmol/L), with an associated peak TSH of 69.0 mU/L (95% CI 44.7–93.2 mU/L).

Only one patient developed clinical symptoms and had the highest observed T4 (T4 60 pmol/L) level and was the only patient to require symptomatic treatment with β-blockade. None of the other patients displayed any classic clinical symptoms of either hyper- or hypothyroid dysfunction. Clinical examination did not reveal any signs of thyroid disease including goitre, exophthalmos, pretibial myxoedema or proximal myopathy.

The hyperthyroid phase in HH spontaneously terminated in all patients without pharmacological intervention with thioamides. Patients were monitored for resolution of their thyroid function, but no recovery was seen. All patients required long-term levothyroxine replacement. Following the management of thyroid dysfunction, all patients continued with ICI immunotherapy. However, thyroid dysfunction did result in a treatment break with ICIs, the longest of which was for 20 weeks (mean 2; range 0–20).

### Associations with thyroid dysfunction

#### Gender

Females were more commonly affected than males, with 11/16 being women (*P* = 0.04), but most notably all patients developing DN hypothyroidism without a preceding hyperthyroid phase were female in this series. Of note, within the total treated population, 57/103 were male and 46/103 were female, thus the female predominance of thyroid dysfunction did not reflect the gender split of the treated population overall.

#### Pre-treatment TSH levels

The pre-treatment mean TSH level in the DN group (5.77 mU/L; 95% CI 2.45–9.09 mU/L) was higher (*P* = 0.009) than in the HH group (2.49 mU/L; 95% CI 1.78–3.22 mU/L) (Supplementary Fig. 1). Patients with a known diagnosis of thyroid dysfunction were excluded from evaluation but we did include patients (*n* = 2) with sub-clinically elevated TSH levels at baseline. Both of these patients went on to develop DN hypothyroidism without a hyperthyroid phase. It is unclear as to whether these patients would have gone on to develop clinical hypothyroidism with depressed T3/T4 levels in the absence of checkpoint blockade. All the patients included in this analysis had normal T4/T3 levels at baseline prior to treatment with immunotherapy.

#### Thyroid autoantibodies

Of the 16 patients identified to have thyroid dysfunction, 12 had thyroid autoantibodies assessed. Five out of the 12 were found to have positive thyroid antibodies (anti-TPO, anti-Tg and anti-TRAb). One patient developed HH on pembrolizumab monotherapy and had a positive anti-TPO titre of 60.43 IU/mL. A second patient developed HH on combination immunotherapy and had a positive anti-TPO titre of 505 IU/mL and was anti-Tg Elisa positive with a concentration of 275.12 U/mL. A third patient who developed DN on pembrolizumab monotherapy had a positive anti-TPO titre of 183 IU/mL and a TRAb titre of 3.5 U/L despite being hypothyroid and requiring levothyroxine. A further two patients who both developed the HH pattern of dysfunction, one receiving pembrolizumab monotherapy and the other combination ICI, had positive anti-Tg antibodies with titres of 208.43 U/mL and 117.95 U/mL, respectively. TRAb and anti-TPO antibodies were not detected in these patients. The remaining seven patients had negative TRAb, anti-TPO and anti-Tg antibodies. There was no correlation with the clinical pattern.

## Discussion

In this report of patients with metastatic malignant melanoma receiving ICI therapy, we have undertaken longitudinal characterisation of patients who developed thyroid dysfunction and shown two clinical patterns of thyroid dysfunction: hyperthyroidism followed by hypothyroidism and *de novo* development of hypothyroidism. We also showed an association with gender and variable presence of autoantibodies. A recent systematic review of 38 randomised clinical trials showed that the risk of thyroid dysfunction was highest with combination therapy, followed by PD1 inhibitors (16). Our analysis shows that the time to onset may also be shorter with combination therapy when compared with monotherapy. The time to onset with monotherapy varies with the longest time interval to onset being 27 weeks within our patient cohort. This highlights the need for continued vigilance throughout the treatment course. While in clinical trials (Supplementary Table 1) the predominant pattern of toxicity was hyperthyroidism transient thyroiditis has been previously identified (13, 17); however, with regular patient sampling throughout treatment with ICIs, we have illustrated that the majority of patients actually experience the transient hyperthyroidism with the HH pattern, with only a minority of patients developing DN hypothyroidism as the initial manifestation of thyroid toxicity.

While the effector mechanisms responsible for the efficacy of ICIs are becoming better understood, the pathophysiological mechanisms underlying irAEs remain to be fully characterised. Previous data has shown that the thyrotoxic pattern is associated with increased ^18^FDG uptake compatible with an inflammatory process (18). It is possible that the threshold for developing autoimmune thyroid disease (AITD) varies in the population and that the use of ICIs lowers the threshold further allowing those who are most susceptible to develop thyroid dysfunction while undergoing therapy. Consistent with this, there is considerable similarity between the thyroid dysfunction associated with ICI use and painless thyroiditis (PT), a subtype of AITD (19, 20). The presentation can be variable; approximately 30% of patients manifest a triphasic pattern (hyper- followed by hypo- and then euthyroidism), while the remaining 70% manifest either the hyper- or hypo- phases in isolation prior to a return to euthyroidism (21, 22). In our patients, we did not see a return to euthyroidism, although that has been reported to occur in a minority of patients (23). The higher risk in women in our patients is consistent with the gender difference seen in painless thyroiditis. While baseline TSH does not appear predictive for ICI-induced thyroid dysfunction (12), it may be indicative of which pattern of dysfunction occurs, since those who developed DN in our series had significantly higher baseline TSH levels than those with the HH pattern. This suggests that some patients may have subclinical thyroid dysfunction at baseline which is then exacerbated by ICI therapy. Although we observed this in two patients (50%), we acknowledge that our sample size is small, and further studies to determine whether baseline TSH levels are truly predictive will need to be prospectively evaluated in a larger patient population. It is likely that both HH and DN patterns of thyroid dysfunction result from a degree of thyroiditis with glandular destruction; however, within the DN cohort, the degree of inflammation does not appear to lead to significant thyroxine discharge. The reasons for this are unclear at this stage but may be due to existing subclinical glandular insufficiency, lower absolute levels of inflammation or different inflammatory mediators.

In painless thyroiditis, it is known that patients have very high levels of anti-TPO antibodies (19). By contrast, we did not find a consistent pattern of thyroid autoantibodies in the patients on ICI with thyroid dysfunction. While Osorio* et al*. found that 8/10 patients in their cohort had positive thyroid autoantibodies (13), this has been less clear in other studies (12, 17). Our case series reinforces the fact that the mechanism of thyroid dysfunction is not clearly related to autoantibodies and alternative, probably cellular-mediated mechanisms may be responsible. It is possible that the presence of autoantibodies is more likely to be a secondary effect of thyroid destruction, possibly by T cells, although this will need further study using tissue sections.

In conclusion, we have characterised the course of patients who developed thyroid dysfunction after receiving ICIs either as monotherapy or in combination. Despite the limitation of this study being a single centre, retrospective review of a small number of patients, there was robust and standardised measurement of biochemical thyroid function allowing consistent characterisation. This illustrates that, unlike the apparent clinical picture reported in clinical trials (1, 2, 3, 4, 5, 6, 7, 8, 9, 10), the majority of patients develop a preceding hyperthyroidism prior to the hypothyroid phase. The levels of T4 can be significant but, despite this, treatment other than β-blockade for symptomatic control is not required. The hyperthyroid phase predictably self terminates with loss of thyroid capability. Furthermore, the natural course of the thyroid dysfunction seems to be permanent. Larger, multicentre studies are needed to further validate these findings. Irrespective of further studies, it seems clear that patients should be monitored for thyroid dysfunction throughout their course of therapy because of the variable temporal relationship, so that symptomatic management of the hyperthyroid phase and timely institution of hormone replacement in the hypothyroid phase can be achieved, optimising symptomatic management and limiting the patient impact of thyroid irAEs.

## Supplementary Material

Figure S1: Box and whisker plot illustrating pre-treatment TSH levels in both the HH and DN patterns of thyroid dysfunction

Figure S2: Classical Triphasic pattern of thyroiditis

Table S1: Incidence of Hyperthyroidism and Hypothyroidism identified in the commercial trials of oncological PD-1 checkpoint inhibitors in the treatment of metastatic malignant melanoma.

## Declaration of interest

Dr Anna Olsson-Brown has received honoraria for speaking at educational events from Roche Pharma, Bristol-Myers Squibb and MSD. She has received honoraria for attending Bristol-Myers Squibb advisory boards. Dr Rosemary Lord has received honoraria from Tezzaro and AstraZeneca. Dr Joseph Sacco has received honoraria for speaking at educational events from Bristol-Myers Squibb. He has received honoraria for attending Bristol-Myers Squibb, Immunocore and Pierre Fabre advisory boards. Dr Jonathan Wagg is an employee of Roche Pharma and holds shares in the company. Prof Mark Coles has received educational honoraria from Roche Pharma. Prof Sir Pirmohamed is the North West England Medical Research Council Fellowship Scheme in Clinical Pharmacology and Therapeutics fellowship scheme leader. Prof Mark Coles has received educational honoraria from Roche Pharma.

## Funding

Anna Olsson-Brown is an MRC clinical training fellow based at the University of Liverpool supported by the North West England Medical Research Council Fellowship Scheme in Clinical Pharmacology and Therapeutics, which is funded by the Medical Research Council (Award Ref. MR/N025989/1), Roche Pharma, Eli Lilly and Company Limited, UCB Pharma, Novartis, the University of Liverpool and the University of Manchester.
