# Supplementary figures and images for: Two distinct clinical patterns of checkpoint inhibitor-induced thyroid dysfunction
Source: Endocr Connect. 2020 Mar 11;9(4):318–25. doi: 10.1530/EC-19-0473 (PMC7159260; doi:10.1530/EC-19-0473)

Baseline pre-treatment TSH mU/L

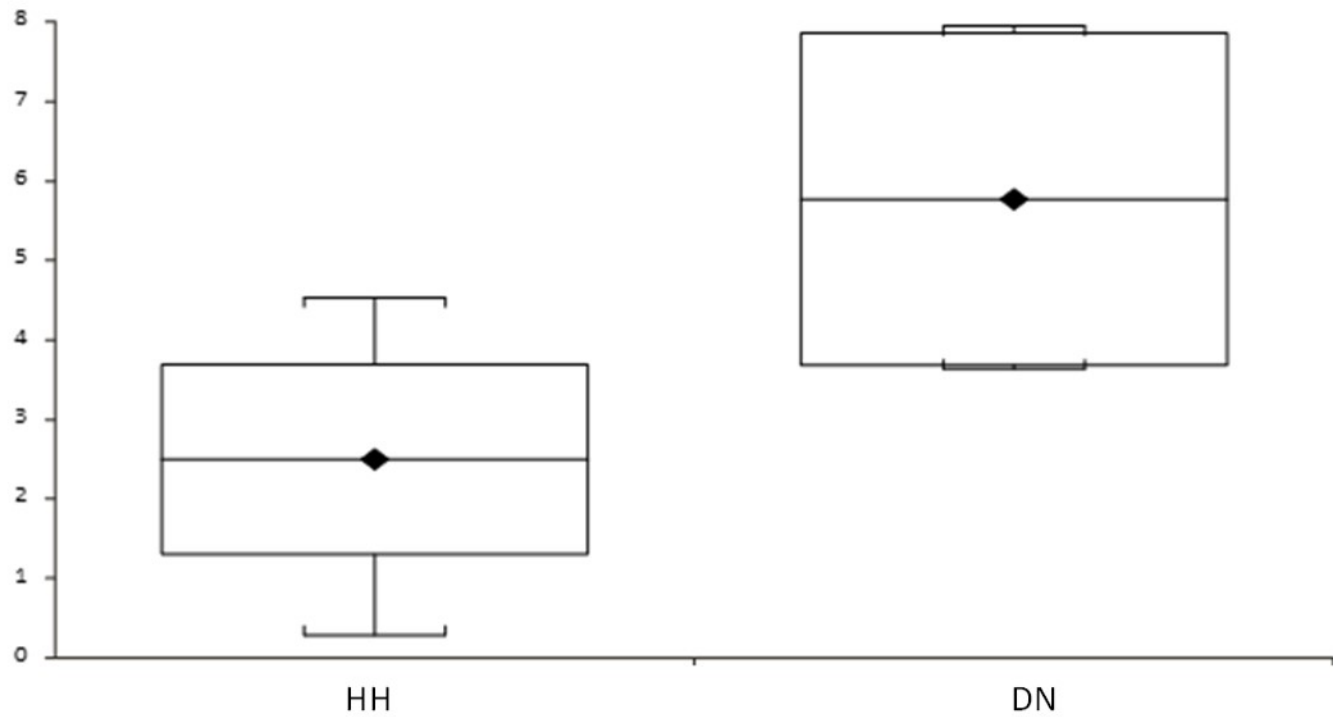

Supplement: Figure S1: Box and whisker plot illustrating pre-treatment TSH levels in both the HH and DN patterns of thyroid dysfunction [file supplementary_figure_1.pdf]

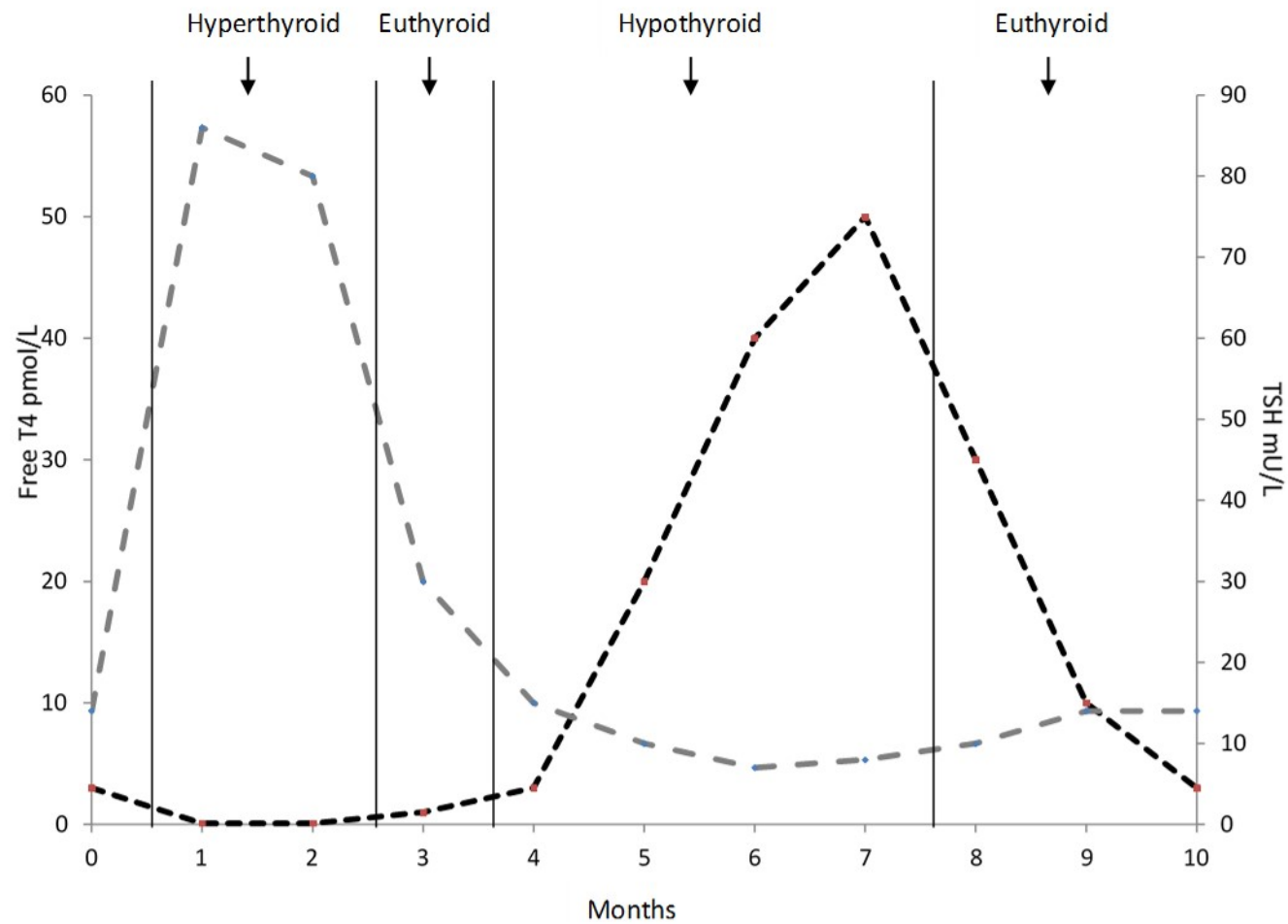

Supplement: Figure S2: Classical Triphasic pattern of thyroiditis [file supplementary_figure_2.pdf]
