# Supplementary material for: Two distinct clinical patterns of checkpoint inhibitor-induced thyroid dysfunction
Source: Endocr Connect. 2020 Mar 11;9(4):318–25. doi: 10.1530/EC-19-0473 (PMC7159260; doi:10.1530/EC-19-0473)
Supplement: Table S1: Incidence of Hyperthyroidism and Hypothyroidism identified in the commercial trials of oncological PD-1 checkpoint inhibitors in the treatment of metastatic malignant melanoma. [file supplementary_table_1.pdf]

Table S1: Incidence of Hyperthyroidism and Hypothyroidism identified in the commercial trials of oncological PD-1 checkpoint inhibitors in the treatment of metastatic malignant melanoma.

Trial	Robert et al (2015)[4]	Weber et al * (2015)[7]	Larkin et al (2015)[1]	Postow et al**[3]	Weber et al***(2016)[9]	Robert et al (2014)[3]	Ribas et al (2012)[12]	Robert et al (2015)[6]	Long et al (2017)[8]
Synonym	Checkmate 066	Checkmate 037	Checkmate 067	N/A	Checkmate 064	Keynote001	Keynote002	Keynote 006	Keynote029
Design	Double Blind Randomised Phase 3 1:1	Open Label Randomised Phase 3 2:1	Double Blind Randomised Phase 3 1:1:1	Double blind Randomised Phase 2 2:1	Open Label Phase 2 1:1	Open Label Phase 1	Randomised Phase 2 1:1:1 design	Randomised Phase 3 1:1:1 design	Open Label Phase 1b
Enrolment	418	631	945	142	140	173	540	834	153
Drug Regime	Nivolumab 3mg/kq q2wkly with dacarbazine matched placebo q3wkly (**1**) **OR** Dacarbazine 1000mg/m2 q3wkly with nivolumab matched placebo q2wkly (**2**)	Nivolumab 3mg/kq q2wkly (**1**) **OR** Investigators Choice of Chemotherapy q3wkly (Dacarbazine/ Paclitaxel and Carboplatin) (**2**)	Nivolumab 3mg/kg q2wkly plus placebo(**1**) **OR** Nivolumab 1mg/kg q3wkly plus Ipilimumab 3mg/kg q3wkly for 4 doses then nivolumab 3mg/kg q2wkly(**2**) **OR** Ipilimumab 3mg/kg q3wkly plus placebo (**3**)	Ipilimumab 3mg/kg q3weekly for 4 doseswith Nivolumab 3mg/kg q3wkly **THEN** Nivolumab 3mg/kg q2wkly (**1**) **OR** Ipilimumab 3mg/kg q3weekly for 4 doseswith Placebo q3wkly **THEN** Placebo 2wkly (**2**)	Nivolumab 3mg/kg q2wkly for 6 doses then Ipilimumab 3mg/kg q3wkly for 4 doses (**1**) **OR** Ipilimumab 3mg/kg q3wkly for 4 doses then Nivolumab 3mg/kg q3wkly for 6 doses (**2**)	Pembrolizumab 2mg/kg q3wkly (**1**) **OR** Pembrolizumab 10mg/kg q3wkly (**2**)	Pembrolizumab 10mg/kg q3wkly (**1**) **OR** Pembrolizumab 2mg/kg q3wkly (**2**) **OR** Chemotherapy (Paclitaxel + Carboplatin/ Paclitaxel/ Carboplatin/ dacarbazine/ oral temozolamide) (**3**)	Pembrolizumab 10mg/kg q2wkly (**1**) For 2/1 **OR** Pembrolizumab 10mg/kg q3wkly (**2**) for 2/1 **OR** Ipilimumab (**3**) 3mg/kg for 4 doses	Pembrolizumab 2mg/kg q3wkly for 2/1 **AND** Ipilimumab 1mg/kg q3wkly for 4 doses
Hyperthyroidism Any Grade N(%)	1: 7(3.4)<br>2: 0(0)	1: 5(1.9)<br>2: 0(0)	1: 13(4.2)<br>2: 31(9.9)<br>3) 3(1)	1: 6(4.3)<br>2: 0(0)	1: Not Stated<br>2: Not Stated	1: 1 (1.1)<br>2: 2 (2.4%)	1: 7(4)<br>2: 2(1)<br>3: 0(0)	1: 18 (6.5)<br>2: 9(3.2)<br>3: 6 (2.3)	16 (10.5)
Hyperthyroidism Severe Grade N(%)	1: 0(0)<br>2: 0(0)	1: 0(0)<br>2: 0(0)	1: 0(0)<br>2: 0(0)<br>3: 0(0)	1: 0(0)<br>2: 0(0)	1: Not Stated<br>2: Not Stated	1: 0(0)<br>2: 0(0)	1: 0(0)<br>2: 0(0)<br>3: 0(0)	1: 0 (0)<br>2: 0 (0)<br>3: 1 (0.4)	2 (1)
Hypothyroidism Any Grade N(%)	1: 9(4.4)<br>2: 1(0.5)	1: 15 (5.6)<br>2: 0(0)	1: 27(8.6)<br>2: 47(15)<br>3: 13(4.2)	1: 15 (16)<br>2: 0(0)	1: 15 (22.1)<br>2: 15 (21.4)	1: 5(5.6)<br>2: 2(2.4)	1: 11(6)<br>2: 15(8)<br>3: 1(0.5)	1: 28 (10.1)<br>2: 24 (8.7)<br>3: 5 (2)	24 (16)
Hypothyroidism Severe Grade N(%)	1: 0(0)<br>2: 0(0)	1: 0(0)<br>2: 0(0)	1: 0(0)<br>2: 1(0.3)<br>3: 0(0)	1: 7(15)<br>2: 0(0)	1: 0(0)<br>2: 0(0)	1: 0(0)<br>2: 0(0)	1: 0(0)<br>2: 0(0)<br>3: 0(0)	1: 1 (0.4)<br>2: 0 (0)<br>3: 0(0)	0(0)

Where no duration is stated the regime duration was until progression/toxicity * /*** Weber et al recorded the incidence of patients with an increased blood thyroid stimulating hormone** Postow et al also recorded the incidence of patients with a decreased blood TSH
